# Stimulus-locked auditory information facilitates real-time visuo-motor sequence learning

**DOI:** 10.3758/s13423-023-02378-z

**Published:** 2023-09-21

**Authors:** Ziyan Han, Daniel Sanchez, Carmel A. Levitan, Aleksandra Sherman

**Affiliations:** 1 Department of Cognitive Science, Occidental College, Los Angeles, CA USA; 2 Department of Psychology, Northwestern University, Evanston, IL USA; 3 SRI International, Menlo Park, CA USA

**Keywords:** Multisensory learning, Visuo-motor learning, Sequence learning, Serial reaction time task, Sensory redundancy, Feedback strategy

## Abstract

Prior research investigating whether and how multisensory information facilitates skill learning is quite mixed; whereas some research points to congruent information improving learning, other work suggests that people become reliant on the redundant information, such that its removal ultimately detracts from the ability to perform a unisensory task. We examined this question using the Serial Interception Sequence Learning (SISL) task, a visuo-motor paradigm in which participants implicitly learn a sequence embedded in noise. We investigated whether adding auditory information in different ways would enhance real time sequence learning and whether any benefits of multisensory learning would persist with visual-only testing. Auditory information was used either as feedback on the visuo-motor task (Experiments 1 and 2) or was presented synchronously with visual information during learning (Experiment 3). Robust sequence-specific performance advantages occurred across conditions and experiments; however, auditory information enhanced real-time performance only when it was synchronized with visual information. Participants were significantly more accurate, faster, and more precise with stimulus-locked auditory information during training. Notably, these benefits did not generalize to the visual-only context, suggesting that the benefits of stimulus-locked auditory information are primarily useful only when the perceptual information is present.

## Introduction

Mastering a procedural skill, such as playing a musical instrument, is a highly dynamic experience requiring the coordination of sensory, motor, and perceptual systems. On the one hand, when multiple sources of congruent sensory information are present, encoding redundancies could potentially facilitate learning and retention of visuo-motor skills. Some past research is consistent with this idea that auditory information can improve visuo-motor learning performance. For example, Finney and Palmer (2003) asked pianists to learn a piece from notation, with and without auditory feedback. Performance on subsequent test trials was better when the piece was learned in the presence of auditory feedback, regardless of whether auditory feedback was present during testing. They also asked pianists to perform pieces that they already knew well (and had presumably learned while hearing themselves play). Notably, error rates were similar when the pieces were performed with or without auditory feedback. Similarly, previous research using a serial-reaction time (SRT) paradigm, in which participants respond with a corresponding button-press when a visual stimulus is presented at a specific location, has shown that auditory information can improve learning. For example, participants’ reaction time improved when tones were mapped onto corresponding response locations (Hoffmann et al., 2001). Auditory information also enhances task performance in perceptual learning paradigms, particularly if the additional information is task-relevant (e.g., Kim et al., 2008; Seitz et al., 2006). In their study, van Vugt and Tillmann (2015) found that participants who learned to tap with auditory feedback that was presented synchronously with their keystrokes improved in their tapping regularity such that they had more consistent spacing between keystrokes. They maintained this improvement in tapping regularity even when auditory feedback was removed, suggesting that multisensory learning may persist in unisensory contexts.

On the other hand, the specific way multisensory information is presented can change the effect of learning performance and retention. The guidance hypothesis, for instance, suggests that while feedback during training boosts real time learning, later performance of a learned task worsens when the feedback cue is removed (e.g., Salmoni et al., 1984); this has been shown in many studies of motor learning (e.g., Maslovat et al., 2009; Park et al., 2000; Winstein et al., 1994). Looking specifically at the effects of visual and auditory feedback on motor learning, Ronsse et al. (2011) found mixed evidence for the guidance hypothesis. Participants learned a bimanual motor task that required challenging coordinated movement across the two hands, with one hand leading the other in performing a pattern. After learning with auditory feedback, there was no performance decrement when feedback was removed. However, performance was significantly worse when visual feedback was removed for those who learned the motor pattern with visual feedback alone, despite the fact that they learned the pattern more quickly than those who learned with auditory feedback only. In this case, the guidance hypothesis applied to visual, but not auditory feedback.

Moreover, research suggests that redundant sensory information, in general, is not necessarily beneficial during learning. Abrahamse et al. (2012) added redundant visual information into SRT sequence learning by arbitrarily mapping cues onto different colors or shapes. There were no benefits observed in sequence learning by providing redundant sensory information. Explicitly learning the order of the sequence benefited error reduction, but also interfered with performance speed (Tanaka & Watanabe, 2017). Similarly, Abrahamse et al. (2009) did not observe benefits by adding congruent tactile information to visual stimuli during sequence learning.

We extended previous research by assessing whether the way in which auditory information was presented would differentially affect visuomotor sequence learning. We added sound in several ways to the Serial Interception Sequence Learning (SISL) task, which requires participants to learn both order and the relevant temporal structure of a sequence (Gobel et al., 2011). SISL is an implicit sequence learning task in which a trained sequence is embedded in pseudorandom noise sequences and is repeated over the course of several training blocks. Even when participants report being unaware of the repeated training sequence, robust learning occurs, reflected in increased accuracy to items that are trained relative to responses to random items. Explicit awareness that a sequence was embedded during training does not impact learning, likely because SISL performance relies on temporal accuracy, which does not seem to be impacted by explicit knowledge (e.g., Sanchez & Reber, 2013). Moreover, because SISL relies on temporal accuracy and general task demands adapt to individual differences on performance, it is well suited to test the impacts of auditory information on visuomotor learning, and considerably more challenging than the standard SRT, thus better reflecting real-world skill learning. SISL has also been previously used to quantify transfer of visual perceptual-motor components (Sanchez et al., 2015) and to demonstrate auditory-driven learning (Han & Reber, 2021). To our knowledge, this is the first study investigating auditory contextual information in visuo-motor sequence learning, focusing on multisensory information integration that employs SISL.

We specifically examined visuo-motor sequence learning performance when tones were presented *as response-locked feedback* to a visually guided sensorimotor task (Experiments 1 and 2), as they might be when playing an instrument like piano or guitar, and when tones were presented in a *stimulus-locked* way with visual information to guide sensorimotor performance (Experiment 3). We subsequently assessed whether learning would persist when auditory information was removed during the test phase.

Our key predictions for the training phase were based on the hypothesis that multisensory information would enhance learning. We expected that adding the auditory information (whether as feedback or as synchronous information) would lead to improved sequence learning relative to learning the same sequences in a visual-only context. Thus, our key predictions for the training phase could manifest as (1) main effects of training condition, which would demonstrate that multisensory information enhances sequence-specific performance on the task, and/or as (2) interactions between training condition and block, which would indicate multisensory context influences the temporal dynamics of performance, such as multisensory information leading to more rapid learning.

However, because previous research is inconsistent, we did not have directional predictions for the testing performance phase of the study. When auditory information is present, it may be beneficial during both real-time learning and in the subsequent test phase (as in Finney & Palmer, 2003), or it may have little usefulness (as in Abrahamse et al., 2009, 2012), or could potentially be useful during learning but hinder test performance (e.g., if the guidance hypothesis holds, even if learning is robust and improved with auditory information, performance would worsen when auditory information is removed). Furthermore, different forms of auditory information (as response-locked feedback versus as stimulus-locked information about the stimulus) could differentially impact performance. In a series of experiments, we explored the impacts of different learning conditions.

## Experiment 1

We began our investigation of whether the addition of auditory information facilitates visuo-motor sequence learning by adding response-locked auditory feedback to the SISL task, adapted from Sanchez et al. (2010). We reasoned that auditory feedback that included an indication of accuracy was the most likely auditory context to show multisensory facilitation in the SISL task because it reflects real-world multisensory learning (e.g., when playing an instrument).

## Method

### Participants

All participants provided informed consent to participate in our study. Participants received either monetary compensation ($15 total for participating in two sessions), or received extra credit via Occidental College’s research participation pool. All materials were approved by Occidental College’s IRB. Sixty Occidental College undergraduate students participated, 52 participants finished both sessions (41 female, 11 male; *M*_age_ = 19.77 years; 6 self-identified as left-handed, 46 right-handed). Two participants were excluded from the explicit knowledge analyses because of missing responses to the confidence rating questions. Participants in each experiment were unique (e.g., participation in Experiment 1 precluded participants from participating in Experiments 2 or 3.).

### Apparatus

The task was programmed with Java, and all parameters can be found on our GitHub page. It was administered on a Dell desktop with a Dell 24-in. monitor (model P2417H) at a 60-Hz refresh rate. Participants sat at a comfortable distance from the monitor such that they could reach the keyboard on the table holding the computer. Sound was presented through Sennheiser Pro headphones at a comfortable, fixed volume for all participants.

### General procedure

To increase statistical power, we employed a within-subjects design. Participants completed two sessions, conducted on different days occurring at least 48 hours apart. Participants were randomly assigned to either multisensory or visual-only conditions for the first session, and completed the other condition during the second session. Each session consisted of training in one condition (e.g., with auditory feedback), a test in both the training context (e.g., with auditory feedback) and in a new context (e.g., with auditory feedback removed), and an assessment of participant’s confidence of having seen the trained sequence. Experiment 1 was preregistered after data collection began, but prior to conducting any data analysis (https://osf.io/fhb4r).

At the beginning of the first session, participants provided informed consent, read experiment instructions presented on the computer screen and heard verbal instructions from the experimenter. All sessions began with a practice block in the modality congruent with that session’s learning (e.g., visual-only learning featured a visual-only practice block). Participants then completed six additional training blocks. After finishing each block, participants were encouraged to take a break. When they were ready to continue, they readied their fingers on the keyboard, informed the experimenter, and the experimenter pressed “continue” to begin the next block. Participants then completed two test blocks (first in the same context as training, followed by a test in a different context), and a block assessing their confidence having seen the trained sequence. Finally, the experimenter collected demographic information including age, gender, handedness, and musical experience.

### The multisensory SISL task and session structure

**Fig. 1:**
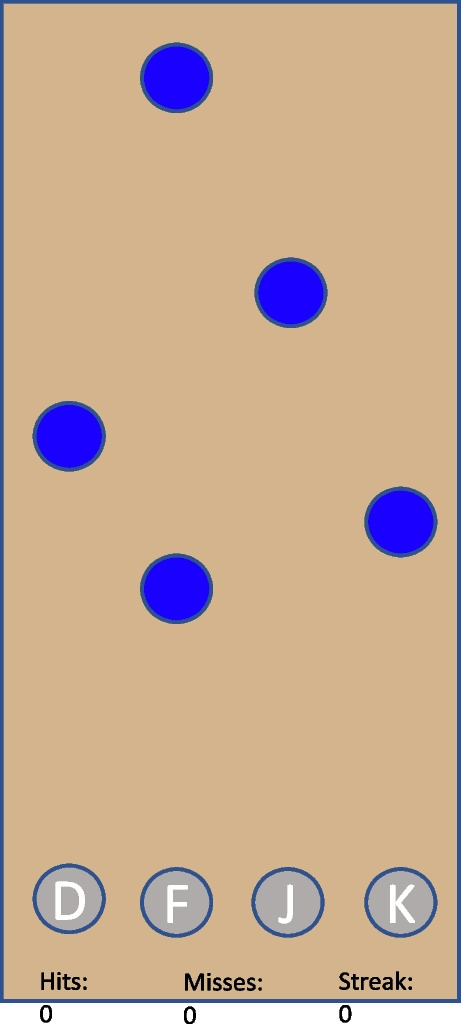
The Serial Interception Sequence Learning (SISL) task is a visuo-motor paradigm in which participants implicitly learn a sequence embedded in noise. The cues (shown in dark blue) fall from top of the screen to the bottom target areas (shown in gray with letters D, F, J, or K). (Color figure online)

In the SISL task, participants pressed a corresponding button when one of four dots moving across the screen fell directly over the corresponding target zone (Sanchez et al., 2010; see https://www.reberlab.org/file/show/SISL?group=667b435bdb923a53 for a live demonstration; Fig. 1). A 12-item second-order conditional (SOC) sequence (*trained sequence*) was embedded in pseudorandom noise sequences (*untrained sequences*) and then repeated over the course of six training blocks. SOC sequences were constructed such that (1) cues were never presented twice consecutively (e.g., D-D would never could occur), (2) pairs of cues were never presented consecutively (e.g., D-F would not be followed by D-F), and (3) a triplet (e.g., D-F-J) was the smallest predictable unit of information, and only occurred once in any given sequence. For example, D-F-K-J-D-K-F-D-K-J-F-D would be an allocable SOC sequence, but D-F-K-J-D-K-F-D-K-J-D-K would not. Untrained noise sequences were constructed by randomly ordering a set of 15 novel 12-item SOC sequences for each participant.

We used the SISL task to measure participants’ accuracy and precision in synchronizing their key presses to cues moving down the screen at different positions. The display consisted of four, unfilled gray rings (“targets”), centered on a horizontal line at the bottom of the display screen. Each target position corresponded to a different letter on a keyboard (from left to right: D, F, J, K). Participants were asked to position their fingers on the home keys of the keyboard such that D corresponded to the left-hand middle finger, F corresponded to the left-hand index finger, J corresponded to the right-hand index finger, and K corresponded to the right-hand middle finger. At the top of the screen were filled blue circles (“cues”) of the same size as the targets, lined up vertically above the targets. The cues fell vertically, landing at the target position.

When the task began, one cue began falling vertically down the screen toward the target zone, subsequently followed by the other cues, with one of two possible interstimulus intervals (ISIs) between each cue (350 ms and 700 ms). The initial time to target was 1,500 ms. Cues began moving at a velocity of 0.3315 pixels/ms. However, since the speed was adaptive, ISI decreased proportionally with increasing speed, so the time between responses is reduced while maintaining the 1:2 timing ratio between sequence items (all code and parameters can be found on our OSF page (https://osf.io/wajex/?view_only=84af845f96a34b24bd996c2c758ba6b6).

Participants were instructed to press the corresponding key when a cue was centered in the target zone. The response was marked as correct if the cue fell within 42.75 pixels of the target zone (both above and below), and the corresponding key was pressed. Incorrect keys, a nonresponse, or pressing multiple keys at once were marked as an error. Participants received both visual and auditory feedback based on accuracy. The cue disappeared and the target turned gray when the response was correct; when the response was incorrect or missed, the cue turned gray, and the target zone turned red.

Performance was assessed every 12 trials, to adjust cue velocity, as needed, to maintain task difficulty and prevent ceiling effects. When accuracy was greater than or equal to 83% (10/12 of the previous cues correct), the speed was increased by 5%; if the accuracy was less than or equal to 50% (6/12 cues), the speed was decreased by 5%, with the slowest possible speed of .25 pixels/ms and the maximum speed of 1 pixels/ms.

### Training phase

Participants completed 2880 total training trials (trials here are defined as a cue crossing the target zone), with six blocks of 40 trial groups; each trial group contains 12 trials. 80% of those trial groups (32) followed the trained sequence and the remaining 8 trial groups followed a pseudorandom sequence. Regular and irregular trial groups were randomly mixed with each other. Participants took a short self-paced break after each block.

Training occurred either in a visual-only context, in which only visual feedback was present during training, or in a multisensory context, in which auditory information was also provided during the six training blocks. Each target position was mapped onto a tone of a different pitch (specifically, 261.63, 293.66, 329.63, and 349.23 Hz). Sound was presented as feedback time-locked to the motor response, such that each corresponding visual target was mapped to a specific pitch, and response-locked auditory feedback was provided such that correct responses led to the correct pitch to sound, whereas incorrect responses were accompanied by an error tone.

### Test phase

Following training, participants performed two test blocks. The first test block matched the training context, and the second test block switched contexts such that multisensory training was followed by a block of multisensory testing and then a block of visual-only testing, while visual learning was followed by a block of visual-only testing and then multisensory testing. During both test blocks, participants were tested on both the trained sequence (120 trials) and on an untrained, novel orthogonal second-order conditional (SOC) sequence (120 trials). Novel SOC sequences were used to assess baseline performance because they balance the predictability and structure of the trained sequence. All novel test sequences were orthogonal to the trained sequence to ensure that they had no shared triplets with the trained sequence that could lead to knowledge transfer (e.g., if a participant was trained on D-F-K, the novel sequence could have D-F-D or D-F-J).

### Explicit knowledge phase

Studies employing SISL typically determine whether participants acquire explicit awareness of the trained sequence by asking participants to rate their confidence on whether a specific sequence was the one they were trained on. Similarly, we asked participants to rate their confidence on the trained sequence (in the same context that they had been trained in), on the two orthogonal sequences they had just been tested on (in the same context that they experienced during training), and on two additional novel sequences (one in a visual context and one in a multisensory context). After each sequence was played twice, participants were asked to indicate “how confident are you that the sequence just presented was the one that repeatedly appeared,” using a scale of 0–4 (0 indicating that the sequence was definitely not the training sequence, 1 indicating that they don’t think it was the training sequence, 2 indicating unsure, 3 indicating that they think it was the training sequence, and 4 indicating that they were sure it was the training sequence). Note that because of our multisession design, and our test phase which occurred in a new sensory context, this measure was somewhat “contaminated” and not as meaningful to interpret as it might be in experiments employing visual-only feedback. Indeed, once participants completed the first session, they were already explicitly aware that there was likely a training sequence in the second session. We included this measure for thoroughness and comparison to other studies but it should be interpreted with caution.

### Data processing and analysis

Participants’ task performance was measured using three metrics: sequence-specific accuracy, cue velocity, and sequence-specific temporal precision. Sequence-specific accuracy was measured for each participant by computing the difference between the percentage of correct responses to the trained sequence and percentage of correct responses to the nonrepeating novel sequence. Cue velocity was used as a metric of task difficulty because the task was adaptive such that the velocity of the cues changed depending on performance accuracy. Cue velocity was determined based on the last velocity recorded at the end of each block. We were also interested in whether multisensory information affected how precise participants’ correct responses were. Because correct responses simply had to be within the target zone (42.75 pixels from the center of the target), participants could be marked correct even if their responses were slightly before or after passing the center of target areas. Thus, we were able to assess temporal precision for each participant by computing the distance of each correct cue response to the center of target and dividing this value by the current velocity of the cue. We then computed the standard deviation, representing how variable participants were relative to their average timing, as we thought it was possible that the addition of auditory information would lead to more consistent motor performance of the temporal precision. Temporal precision for incorrect responses was not assessed as incorrect responses may have different causes making it difficult to draw meaningful inferences (e.g., wrong key press, multiple key presses, low degree of temporal precision). As for sequence-specific accuracy, sequence-specific temporal precision was computed by subtracting precision for untrained sequences from precision for trained sequences.

Because each experiment had several independent variables and dependent variables, we shifted our significance criterion to *p* < .01. All data are available on OSF (https://osf.io/wajex/).

## Results and discussion

Based on prior research, we predicted that participants' performance would improve with practice across all conditions. We conducted 2 × 6 repeated-measures analyses of variance (ANOVAs), with training condition (visuo-auditory feedback, visual-only) and training block (1–6) as within-subjects factors, and sequence-specific accuracy, cue velocity, and temporal precision as dependent measures. There was an increasing sequence-specific performance advantage with training for both accuracy, *F*(5, 255) = 20.156, *p* < .001, η_p_^2^ = 0.28, and cue velocity, *F*(5, 255) = 14.686, *p* < .001, η_p_^2^ = 0.22. Participants more accurately responded to sequence-specific cues at faster speeds during the later training blocks than during earlier training blocks. Sequence-specific temporal precision, however, did not change as a function of block, *F*(5, 255) = 0.853, *p* = .513, η_p_^2^ = 0.02.

**Fig. 2:**
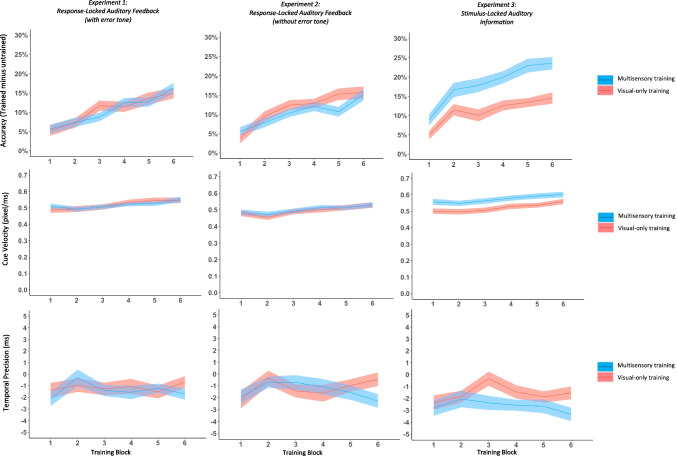
Performance accuracy (trained minus untrained , top), cue velocity at the end of each block (middle), and temporal precision (trained minus untrained, bottom) is shown for multisensory training (blue) and visual-only training (red) for each training block. (Color figure online)

Contrary to our pre-registered predictions, we found no evidence that the addition of auditory feedback affected sequence-specific performance during training for any of the three performance metrics: accuracy, temporal precision, and cue velocity (Fig. 2, *F*s < 0.583, *n.s.*). Performance during all six training blocks was the same irrespective of whether sequences were presented with or without auditory feedback.

**Fig. 3:**
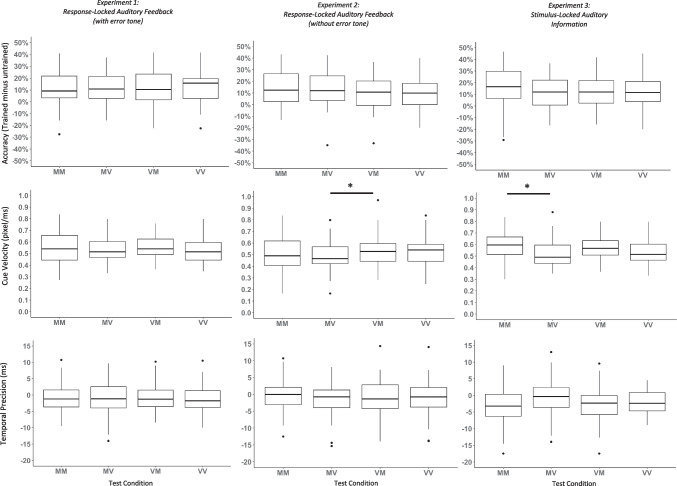
Performance accuracy (trained minus untrained, top), cue velocity at the end of each block (middle), and temporal precision (trained minus untrained, bottom) is shown for the four training-test phases: multisensory training and multisensory test (MM), multisensory training and visual-only test (MV), visual-only training and multisensory test (VM), visual-only training and visual-only test (VV)

Next, we analyzed sequence-specific learning by comparing participants’ test block performance. Note that the primary purpose of the test blocks was to determine whether any benefits of learning in a multisensory context persisted to a visual-only context. To test this, we computed one-way repeated-measures ANOVAs, with training-test condition (MM, MV, VM, VV) as a within-subjects factor and sequence-specific performance accuracy, cue velocity, and temporal precision as dependent measures. However, because there was no evidence for auditory facilitation during training, we did not expect that notable condition-wide differences would emerge during the test phase. Indeed, the one-way ANOVA was not significant for any of the three metrics (Fig. 3, *F*s < 1.906, *n.s.*).

Finally, we determined whether the addition of auditory information increased explicit knowledge of the trained sequence, and if so, whether explicit knowledge had an impact on sequence learning. We conducted a 2 × 2 ANOVA, with training condition (visuo-auditory feedback, visual-only) and sequence type (trained, untrained) as within-subjects factors and participant’s confidence ratings as the dependent variable. Participants reported more confidence with the trained sequence relative to the untrained sequences, *F*(1, 49) = 89.75, *p* < .001, η_p_^2^ = 0.65 (see Table 1). There were no significant differences in confidence ratings for sequences trained with auditory feedback and sequences trained with visual-only information, *F*(1, 49) = 0.084*, p =* .77*,* η_p_^2^ = 0.0017 (see Table 1). As previously discussed, explicit knowledge does not seem to affect performance on the SISL task. To confirm this, we correlated confidence ratings with sequence-specific accuracy and cue velocity at test. In all cases, test performance was unrelated to confidence (at *p* < .01). Neither trained sequence performance accuracy at test, MM: *r*(49) = 0.041, *p* = .772; VV: *r*(49) = −0.175, *p* = .221, nor cue velocity at test were significantly correlated with confidence, MM: *r*(49) = 0.025, *p* = .858; VV: *r*(49) = 0.282, *p* = .045. That is, people who were more confident that they recognized the sequence did not necessarily have performance advantages.

**Table 1:** Confidence ratings for Experiments 1 (top), 2 (middle), and 3 (bottom)

*Confidence Ratings*	*Multisensory contexts*	*Visual-only*
Trained sequence	M_Exp1_ = 3.098, SD_Exp1_ = 1.237 M_Exp2_ = 3.293, SD_Exp2_ = 1.076 M_Exp3_ = 3.098, SD_Exp3_ = 1.345	M_Exp1_ = 2.922, SD_Exp1_ = 1.146 M_Exp2_ = 2.568, SD_Exp2_ = 1.258 M_Exp3_ = 2.392, SD_Exp3_ = 1.524
Untrained sequence	M_Exp1_= 1.618, SD_Exp1_ = 0.846 M_Exp2_ = 1.366, SD_Exp2_ = 0.802 M_Exp3_ = 1.623, SD_Exp3_ = 0.848	M_Exp1_ = 1.673, SD_Exp1_ = 0.693 M_Exp2_ = 1.565, SD_Exp2_ = 0.689 M_Exp3_ = 1.603, SD_Exp3_ = 0.831

One potential reason that we did not find multisensory facilitation is that the auditory feedback contained error tones, which may have been disruptive to learning, especially as the adaptive nature of our task meant that participants were frequently making errors. Because accuracy was already clear to participants through unambiguous visual feedback, the error tones may not have provided useful information, yet they disrupted the experience of hearing a melody.

## Experiment 2

To assess whether auditory feedback would be useful when it was not disrupted by error tones, in Experiment 2 participants simply heard tones corresponding to the keys that they pressed, making the experience more akin to playing a musical instrument, where pressing the wrong key or strumming incorrectly carries the consequence of hearing the wrong melody. In all other ways, Experiment 2 was identical to Experiment 1. We did not create an additional preregistration; all analyses were consistent with our preregistration plan for Experiment 1.

## Method

### Participants

Sixty-one Occidental College undergraduate students participated; 58 participants finished both sessions (49 female, nine male; *M*_age_ = 19.55 years; six self-identified as left-handed, 51 right-handed, one ambidextrous). All participants provided informed consent to participate in our study. Participants received either monetary compensation ($15 total for both sessions), or received extra credit via Occidental College’s research participation pool. Participants in prior experiments were excluded from participation in follow-up experiments. All materials were approved by Occidental College’s IRB.

## Results and discussion

As in Experiment 1, there was an increasing sequence-specific performance advantage with training for both accuracy, *F*(5, 285) = 20.892, *p* < .001, η_p_^2^ = 0.268, and cue velocity, *F*(5, 285) = 19.180, *p* < .001, η_p_^2^ = 0.252. Participants more accurately responded to sequence-specific cues at faster speeds during the later training blocks than during earlier training blocks. Sequence-specific temporal precision, however, did not change as a function of block, *F*(5, 285) = 1.519, *p* = .184, η_p_^2^ = 0.026.

Moreover, we found no evidence that the addition of auditory feedback affected sequence-specific performance during training for any of the three performance metrics: accuracy, temporal precision, and cue velocity (Fig. 2, *F*s < 1.749, *n.s.*). Performance during all six training blocks was the same irrespective of whether sequences were presented with or without auditory feedback. This suggests that auditory feedback, even without error tones, may not be the right auditory context to observe meaningful multisensory facilitation.

Given that we again found no evidence that auditory feedback affected visuo-motor sequence performance, we did not expect to find any test performance effects. Although test performance did not differ between conditions for sequence-specific accuracy, *F*(3, 171) = 2.147, *p* = .096, η_p_^2^ = 0.036, or temporal precision, *F*(3, 171) = 0.228, *p* = .877, η_p_^2^ = 0.004, there was a significant effect of condition for cue velocity, *F*(3, 171) = 3.176, *p* = .026, η_p_^2^ = 0.053. However, this did not reach our more conservative significance criteria (*p* < .01). Moreover, post-hoc *t* tests suggested that the effect was driven primarily by a difference between MV (*M* = 0.497, *SD* = 0.126) and VM (*M* = 0.540, *SD* = 0.130), such that velocity was slower for MV than for VM (Fig. 3). Although this may suggest that performance drops when auditory information is taken away at test, the evidence is not compelling, as we do not see significant differences between MM and MV, a more key comparison for the guidance hypothesis.

For explicit sequence recognition, we conducted a 2 × 2 ANOVA, with training condition (visuo-auditory feedback, visual-only) and sequence type (trained, untrained) as within-subjects factors and participant’s confidence ratings as the dependent variable. Participants were more confident that they saw the trained sequence relative to the untrained sequences, *F*(1, 57) = 80.314, *p* < .001, η_p_^2^ = 0.585 (Table 1). Moreover, participants’ confidence was significantly higher for sequences trained with auditory feedback than for sequences trained with visual-only information, *F*(1, 57) = 6.841, *p* = .01, η_p_^2^ = 0.107 (Table 1). There was also a significant interaction between sequence type and training condition, *F*(1, 57) = 8.220, *p* < .001, η_p_^2^ = 0.126, such that people were most confident that they had previously heard the trained sequence when it was presented in a multisensory context. However, explicit knowledge did not correlate with sequence-specific test accuracy, MM: *r*(56) = 0.008, *p* = .953; VV: *r*(56) = 0.132, *p* = .322, or with cue velocity at test, MM: *r*(56) = 0.005, *p* = .969; VV: *r*(56) = 0.230,* p* = .083. That is, people who were more confident that they recognized the sequence did not necessarily have performance advantages.

Taken together, the results of Experiments 1 and 2 demonstrate that auditory feedback did not enhance learning. While auditory feedback did lead to more recognition of the trained sequence, this had no benefits on participants’ ability to execute the sequence, likely due to the unambiguous visual feedback as well as the challenging nature of the SISL task, which is robust even when participants have explicit knowledge.

## Experiment 3

As there was no boost in performance or learning when auditory feedback was present with or without error tones, we next considered whether a different auditory context might better demonstrate multisensory facilitation. Thus, in Experiment 3, we used stimulus-locked auditory information to determine whether synchronous audiovisual information would enhance learning and performance. Tones were presented synchronously with the visual cue falling over the target zone, regardless of response accuracy. Using another naturalistic analogy, stimulus-locked presentation more closely resembles playing an instrument as part of a band or ensemble, such that one is executing the motor program with the goal of synchronizing to an external source rather than focusing on the auditory feedback of just one’s own individual performance. All task procedures were otherwise identical to Experiments 1 and 2. Hypotheses and planned analyses were all preregistered (https://osf.io/pw2bt).

## Method

### Participants

Fifty-nine Occidental College undergraduate students participated in the study, 52 of whom finished both sessions (39 female, 13 male, *M*_age_ = 19.48; 49 right-handed, one left-handed, two ambidextrous). All participants provided informed consent to participate in our study. Participants received either monetary compensation ($15 total for both sessions), or received extra credit via Occidental College’s research participation pool. All materials were approved by Occidental College’s IRB. Only participants who completed both sessions were included in analyses. One participant was excluded from the explicit knowledge analyses because of a missing confidence rating. Participants in prior experiments were excluded from participation in follow-up experiments.

## Results and discussion

Consistent with previous research and with Experiments 1 and 2, there was an increasing sequence-specific performance advantage with training for both accuracy, *F*(5, 255) = 22.101, *p* < .001, η_p_^2^ = 0.302, and cue velocity, *F*(5, 255) = 13.771, *p* < .001, η_p_^2^ = 0.213. Participants more accurately responded to sequence-specific cues at faster speeds during the later training blocks than during earlier training blocks. Sequence-specific temporal precision, however, did not change as a function of block, *F*(5, 255) = 1.335, *p* = .250, η_p_^2^ = 0.026.

Most crucially, and unlike Experiments 1 and 2, training with stimulus-locked auditory information significantly improved sequence-specific performance on all three performance metrics. Participants were more accurate, *F*(1, 51) = 30.727, *p* < .001, η_p_^2^ = 0.376, maintained significantly faster speeds, *F*(1, 51) = 16.384, *p* < .001, η_p_^2^ = 0.243, and were more temporally precise, *F*(1, 51) = 8.458, *p* = .005, η_p_^2^ = 0.142, while training with stimulus-locked auditory information than with visual-only information (Fig. 2). There were no significant interactions between block and condition for any of the performance metrics (*F*s < 1.612, *n.s.*).

For the test phase, we were interested in assessing whether the benefits of a multisensory context during training persisted to a visual-only context in the test phase. We compared performance between all four conditions (MM, MV, VM, and VV) using a one-way repeated-measures ANOVA. There were no significant (at *p* < .01) sequence-specific accuracy, *F*(3, 153) = 1.713, *p* = .167, η_p_^2^ = 0.032, or temporal precision advantages, *F*(3, 153) = 2.987, *p* = .033, η_p_^2^ = 0.055, between conditions. However, there was an effect on cue velocity, *F*(3, 153) = 7.364, *p* < .001, η_p_^2^ = 0.126.

Key to the guidance hypothesis is what happens when participants who learn with multisensory information no longer have access to auditory cues (MM vs. MV). Participants were unable to maintain as high of speeds once they lost access to auditory information (M_MV_ = .522, SD_MV_ = .113) relative to when they had access to auditory information (M_MM_ = .584, SD_MM_ = .128), *t*(51) = 4.022, Cohen’s *d*= .558, *p*_*Bonf*_ < .001. Consistent with this, test performance was no better when the auditory cue was removed relative to visual-only training and test (Fig. 3, M_VV_ = .542, SD_VV_ = .110). These results support the guidance hypothesis that removing the auditory information that was used during learning hinders test performance.

Participants in Experiment 3 also reported being more confident that they had seen the trained sequence relative to the untrained sequences, *F*(1, 50)= 77.768 , *p* < .001, η_p_^2^ = 0.609. There was no difference in confidence ratings between sequences trained with stimulus-locked auditory information and sequences trained with visual-only information, *F*(1, 50) = 4.183 , *p* = .163, η_p_^2^ = 0.039. There was also a significant interaction between sequence type and training condition, *F*(1, 50) = 7.14, *p* = .01, η_p_^2^ = 0.125, such that people were most confident that they had previously heard the trained sequence when it was presented in a multisensory context. As in Experiments 1 and 2, we correlated confidence ratings with sequence-specific accuracy and cue velocity at test. Neither performance accuracy at test, MM: *r*(50) = −0.031, *p* = .831; VV: *r*(50) = 0.096, *p* = .497, nor cue velocity, MM: *r*(50) = 0.192, *p* = .176; VV: *r*(50) = 0.129, *p* = .363, were correlated with confidence.

## General discussion

Our primary research question asked whether adding auditory information enhances sequence learning performance. Crucially, the nature of the temporal embedding determined whether the information enhanced training performance; sequence-specific accuracy, temporal precision, and speed were enhanced by the addition of stimulus-locked auditory information, but not by the addition of response-locked auditory feedback. The stimulus-locked condition gives participants information about the desired response, while the response-locked condition gives information about one’s performance. While the visual information is available in advance to allow a participant to prepare their motor response, the auditory information is primarily useful in giving additional information about the timing of responses and may enhance learning performance of the sequences. The auditory system tends to be better than the visual system for temporal tasks (e.g., Welch et al., 1986)—and we only saw benefits of adding sounds when auditory information reinforced the desired timing, as opposed to as a cue to letting participants know if they were early/late. Response-locked timing information may not have benefitted learning because visual information about temporal precision was also provided spatially; while audition is typically the better modality for timing, participants were receiving visual feedback about the timing of their keypresses in the form of the overlap between the cue and the target. The visual system is highly sensitive to small spatial differences (e.g., Welch et al., 1986), and thus, auditory feedback may not have given as much added value compared to using auditory information to emphasize the target time.

Conversely, the stimulus-locked information might enhance the ability to learn the trained sequence through more consistent reinforcement of the pattern, as every cue was accompanied by tones. In the response-locked experiments, the auditory sequence was only presented in full when participants were performing it correctly; the adaptive nature of our task was structured to be very challenging, with overall accuracy kept below 83%, meaning a substantial number of tones were absent. Although our results seem to be in contrast with research adding auditory feedback to the SRT task (e.g., Hoffmann et al., 2001), SRT tasks are inherently less challenging. Only participants with high levels of accuracy (>90%) are included in analyses, and inaccurate trials are excluded. In this way, in experiments like Hoffmann et al.’s (2001), there were minimal disruptions to the melodic structure, resembling our stimulus-locked condition more than our response-locked experiments.

Having established that stimulus-locked multisensory information can lead to enhanced learning, we then asked whether these benefits would persist when tested in a visual-only context. In other words, when the auditory information was removed, would participants still retain their enhanced performance on the trained sequence? There was no evidence that the inclusion of auditory information during training enhanced performance once that information was removed during the test; this conclusion is consistent with the guidance hypothesis.

However, participants still showed sequence-specific advantages. Across experiments, performance on the trained sequences was more accurate than performance on untrained sequences, and this held true even for the MV conditions where multisensory training was followed by visual-only testing. Thus, the ability to express knowledge of the learned sequence was not entirely dependent on the auditory information. These results are consistent with those of Sanchez et al. (2015), where participants experienced a context switch, and showed evidence of information transfer that was partial rather than complete.

Although previous research with the SISL task has demonstrated that explicit knowledge is independent of performance on the task (e.g., Sanchez & Reber, 2013), research with other tasks does suggest a role of explicit knowledge (e.g., Wong et al., 2015). Past research with a multisensory version of the SRT task suggests that congruent auditory information may facilitate performance by increasing the probability that participants would develop explicit knowledge of a sequence (Silva et al., 2017). In our study, phenomenologically, participants could experience the sounds as a melody. Stöcker and Hoffmann (2004) suggest that tones may improve the ability to chunk together movements due to the ability to perceive tone sequences as melodies. In our experiments, this melody would be more strongly emphasized in the stimulus-locked condition. However, we did not see significantly higher confidence in rating. We also found no relationship between reported confidence rating and performance on our task metrics. This result is also similar to that of Han and Reber (2021) where performance on an auditory SISL task was generally not influenced by explicit knowledge. However, because we tested confidence after changing context at test (including a condition of visual learning and multisensory testing), our confidence rating task was a contaminated measure, and we cannot rule out the possibility that the sequence became more explicit during the testing. Future work could more directly test explicit knowledge in both the condition of training and in the opposite condition without testing across contexts first.

This work supports the claim that perceptual-motor representations integrate across elements (Abrahamse et al., 2010), even cross-modally. Although transfer does not occur from one modality to another, such as training on visual and testing on auditory (Han & Reber, 2021), when both types of information are present during training, they facilitate learning and expression during a test that features the integrated components. Consistent with the guidance hypothesis, multisensory information can be bound to the representation and this can impact performance when the information is removed.

Why don’t the benefits of stimulus-locked auditory information during learning persist once that information is removed? One possibility is that auditory information is only useful in real time because of the dynamic changes in speed during the experiment. Rather than “hard coding” a learned motor pattern with fixed intervals between key presses, participants must adjust their timing throughout the experiment. Thus, it may be that enhanced performance during stimulus-locked training is from the real-time information about the timing rather than a more enduring internationalization of the pattern.
